# Taking Crohn’s Disease Personally

**DOI:** 10.5041/RMMJ.10111

**Published:** 2013-04-30

**Authors:** Yehuda Chowers

**Affiliations:** Department of Gastroenterology, Rambam Health Care Campus, Haifa, Israel

**Keywords:** Anti-TNF, biomarkers, Crohn’s disease, calprotectin, C-reactive protein, lymphoma, serology, thiopurines

## Abstract

Crohn’s disease (CD) is a heterogeneous disorder that can involve any segment of the gastrointestinal tract. The pathogenesis of CD is unknown but is thought to involve an uncontrolled immune response triggered by an environmental factor in a genetically susceptible host. The heterogeneity of disease pathogenesis and clinical course, combined with the variable response to treatment and its associated side effects, creates an environment of complex therapeutic decisions.

Despite this complexity, significant progress has been made which allows physicians to start and predict disease behavior and natural course, response to therapy, and factors associated with significant side effects.

In this manuscript the data pertaining to these variables including clinical, endoscopic and the various biological and genetic markers are reviewed, and the possibility of tailoring personal treatment is discussed.

## INTRODUCTION

Crohn’s disease (CD) is a heterogeneous inflammatory disorder that may involve any segment of the gastrointestinal tract from mouth to anus. Manifestations of CD are protean, and inflammation can lead to complications such as intestinal strictures, fistula formation, and extraintestinal manifestations including arthritis, skin involvement in the form of erythema nodosum and pyoderma gangrenosum, and ocular complications, as well as various less frequent extraintestinal organ involvement. Other sequelae may be secondary to intestinal loss of function leading to malabsorption. Thus, an array of metabolic disorders such as bone demineralization, nephrolithiasis, and various forms of anemia may occur. Consequently, CD may cause significant morbidity. Moreover, increased mortality was reported by several authors.^1^–^3^ Because of its variable behavior, attempts were made to classify disease in order to adapt treatment accordingly. The most recent and widely used is the Montreal classification which takes into account the age of presentation, involved organs, disease behavior (inflammatory, stricturing, or fistulizing), and whether perianal involvement is present.^4^ The clinical complexity of CD is intensified by the variable frequency of disease flares which have an obvious impact on patient well-being. Cohort studies from recent years have demonstrated that shifting from one disease phenotype to another is frequent during the life course of patients. In one landmark study it was shown that up to 80% of the patients will suffer from a stricturing or penetrating complication over 20 years of follow-up.^5^

The observation of changing disease patterns and accumulation of tissue damage over time suggests that it may be the result of repeated inflammatory activity during flares and hence potentially preventable by administration of appropriate treatment. Although straightforward, this simple logic is difficult to practice when reduced to practical cost–benefit terms, both from the patients’ well-being and actual cost perspectives. Implementation of successful preventive treatment would have to provide effective therapy and assure that side effects and cost are in proportion to clinical efficacy. Establishing such treatment strategy necessitates tools that allow quantification of tissue damage, scaling and quantifying treatment side effects, and, most importantly, delivering care to those who are most likely to benefit from it. The last-mentioned point requires identification of predictive biomarkers to recognize not only patients who will suffer from a progressive disease course but also those who will respond to a given treatment.^6^ Moreover, once these patients are identified, other predictive biomarkers will define those in whom response will actually be associated with tissue damage prevention and among them, those in whom side effects would be tolerable. The substantial variability of disease behavior and drug metabolism and response, combined with our relative ignorance of drug mechanisms of action and long-term effects, make the implementation of this approach a complex task. However, the understanding that improving patient quality of life depends on such treatment has actually changed the way it is perceived with a shift from an emphasis on symptom control to attempts to modify disease course and outcomes.^7^ This understanding has led to efforts for creating the appropriate tools for practicing preventive care and to the understanding that it would have to be tailored and personalized as much as possible. For example, an international task force has recently created a novel MRI-based tool to measure disease damage (as opposed to disease activity)^8^ and a tool to measure patient disability based on international standards.^9^ Availability of such measurements is imperative for assessment of various treatment approaches.

## PREDICTING DISEASE COURSE

Categorization and definition of the various disease phenotypes is a first step for tailoring therapy because treatments can be subsequently matched accordingly. The most available and straightforward approach is the use of clinical parameters. The combined information obtained by these measures is used to identify patients at high risk of a more aggressive disease course. A number of studies examined clinical characteristics and aimed to identify patients at risk for a complicated disease course. For example, Beaugerie at al. defined disabling disease as need for hospitalization, two or more steroid courses, or need for immunosuppressive therapy. They identified risk factors including age <40 at time of diagnosis, presence of perianal disease, and requirement for steroids at first flare as risk factors for a complicated course. The authors noted that a combination of two or three risk factors had a positive predictive value for complicated disease of 0.91 and 0.93, respectively.^10^ These parameters were partially corroborated in other studies.^11^,^12^

Another way to approach this challenge is to probe into disease pathogenesis. Such approach may actually allow tackling the problem from its very beginning. However, the precise pathogenesis of CD is unknown. Nonetheless, during recent years a paradigm of disease pathogenesis has emerged in which it is envisioned that CD is caused by an environmental insult in a genetically susceptible host which results in an inappropriate immune response that in turn leads to tissue damage.^13^ Of these, the more tangible component is the genetic background.

The first and very significant insight into the genetic background of CD has been published in 2001 when two groups independently reported on the association of CD with *NOD2/CARD15*.^14^,^15^ Three *NOD2* polymorphisms have been associated with up to 40% of CD patients in Western populations. However, these polymorphisms are absent in the Asian CD patient population, and other genetic polymorphisms seem to be involved in disease pathogenesis of these patients.^16^ Other major genetic associations described were with the autophagy pathway^17^ and the IL-23 receptor genes.^18^ There appears to be some interaction between the different relevant genetic associations. For example, the NOD2 protein and ATG16L1 co-localize at bacterial entry location, a function which appears to be altered in cases of a NOD2 frame shift mutation.^19^ These observations suggest that genetic variability in mechanisms of processing and presentation of bacterial antigens to the gut innate immune system are important in the pathogenesis of CD.

It is notable that all major pathways implicated by genetic studies to be involved in CD pathogenesis seem to be involved in multiple physiologic processes, and their exact role in disease pathogenesis is not clear. Hence, alteration in NOD2 was suggested to poorly regulate TLR2 signaling,^20^ to be associated with defective mucosal defens in secretion,^21^,^22^ and to lead to unregulated IL-1β secretion.^23^ Despite the fact that CD presents as an immune mediated disorder, i.e. tissue damage is caused by overactivation of the immune system, later studies have suggested that NOD2 polymorphisms may be associated with a reduced inflammatory response.^24^ Similarly, autophagy is involved in many cellular processes including an anabolic function, antigen presentation, and handling of intracellular bacteria.^25^ The IL-23 pathway is no exception. IL-23 has a role in maturation of IL-17-secreting cells and was shown in animal models to be of major importance in mediating intestinal inflammation.^26^ Furthermore, blocking the p40 subunit of IL-23 (and also IL-12) by monoclonal antibodies was shown to be efficacious in clinical studies.^27^ Thus, intuitively one can assume that the protective polymorphism in the IL-23 receptor results in down-regulation of a proinflammatory response. However, a trial in which anti-IL-17, the downstream cytokine of IL-23, was blocked was negative, and signs for exacerbation of inflammation could be detected.^28^ In hindsight, these results are not completely surprising in view of two studies in which intestinal (as opposed to peripheral) IL-17-secreting cells were shown to have a suppressor phenotype both in mice^29^ and in humans.^30^

Taken together, the function of the major three pathways that were associated with CD by genetic studies is variable and can lead to many plausible disease mechanisms and hence a clear paradigm by which the disease can be categorized and the pathogenic mechanisms elucidated and targeted is still not in hand. Furthermore, the different genetic background of Asian and Western hemisphere CD patients may suggest that CD is not a disease that results from one mechanism, but rather a syndrome in which the various clinical outcomes represent a pattern of response to different pathogenic pathways. Therefore, it is not surprising that an attempt to categorize CD patients according to their genetic background was only marginally successful^31^ and does not provide the needed predictive assay. A further hint of the reasons underlying the difficulties in classifying CD according to the genetic background may be that over 160 loci have been associated with inflammatory bowel diseases (both CD and ulcerative colitis), of which many overlap. This type of overlap may be even more apparent when only colonic CD is considered.^32^–^34^

Additional modalities may be used as markers to categorize CD:

- **Endoscopy:** Endoscopy offers the opportunity to observe the diseased organ directly. In order to be useful, standardized scales and definitions were developed. The CD endoscopic index of severity (CDEIS)^35^ and the simple endoscopic index of severity (SES-CD)^36^ were developed and validated. The predictive value of colonic endoscopic findings was demonstrated in a study which showed that in colonic CD severe endoscopic lesions were associated with increased risk of colectomy.^37^ Similarly, severe post-surgical ileal mucosal lesions were associated with worse outcome.^38^ However, more data are needed to substantiate these observations and include them in an algorithm for selecting treatment.
- **Biomarkers:**C-reactive protein (CRP) is considered a marker of inflammation, and elevated CRP levels correlate with active disease.^39^,^40^ CRP was also used as a predictor for later surgery in CD ileitis patients.^41^ CRP levels were found to be predictive for long-term treatment response both as a predictor of relapse after cessation of azathioprine treatment^42^ and for maintenance of response in infliximab-treated patients.^43^,^44^ However, not all patients respond equally with elevated CRP to inflammation. For example, in one study it was demonstrated that the 717 mutant homozygote and heterozygote status in the CRP-encoding gene was associated with lower CRP levels,^43^ and in another study up to 30% of patients with active inflammation did not have elevated CRP levels.^45^ Fecal calprotectin is another marker of intestinal inflammation that is increasingly used in clinical practice. It was shown to correlate with intestinal inflammation^46^ and to predict clinical relapse,^47^ although it was shown to be less useful for ileal CD.^48^ In a recent meta-analysis of 672 patients (of whom 354 had CD) fecal calprotectin was 78% sensitive and 73% specific, with ROC of 0.83, in predicting relapse in quiescent inflammatory bowel disease (IBD).^49^ Thus, inflammatory surrogate markers can assist in determining the presence of active inflammation, long-term risk of surgery, and risk of relapse. However, more studies are needed to substantiate these observations, and the ability to rely on these markers is not inclusive of all patients.
- **Serology:** A number of studies have demonstrated that CD patients develop antibodies against various microbial antigens. Studies have demonstrated patterns of antibody responses to be associated with specific CD patient characteristics. Thus, in one study, anti-CBir1 antibodies (against Escherichia coli flagellin) were associated with fibrostenosis, internal penetrating disease, small bowel involvement, and surgery. Interestingly, a possible link to genetic predisposition was suggested by the demonstration that titers of anti-CBir1 were significantly higher in patients with CD carrying at least one NOD2 variant as compared to those carrying no variant.^50^ In an additional study the investigators tested the association of three microbial-related antibodies with clinical patient characteristics. They demonstrated that patients expressing anti-Pseudomonas bacterial component (I2) antibodies were more likely to have fibrostenosing disease and to undergo small bowel surgery, and that patients with anti-Escherichia coli outer membrane porin C (OmpC) were more likely to have internal perforating disease and also underwent more small bowel surgery. Patients positive for I2, OmpC, and anti-Saccharomyces cerevisiae (ASCA) were the most likely to need small bowel surgery (72.0%; odds ratio 8.6; *P*< 0.001) compared with patients without such reactivity (23.0%).^51^ The association of anti-microbial antibodies with disease phenotypes was further extended and was shown to predict disease behavior. Positive serology for anti-glycan antibodies gASCA, AMCA, ACCA, and Anti-L predicted a faster progression to a severe disease course.^52^

Recently, a novel approach for predicting disease behavior was published. The investigators prospectively performed gene expression analysis in CD8+ cells obtained from CD and ulcerative colitis(UC) patients and followed patients up to 700 days (albeit in small numbers). They were able to demonstrate that transcriptional profiling allowed prediction of an aggressive disease course and that this method was superior to ASCA positivity and clinical parameters.^53^

In summary, clinical, serologic, genetic, and functional data are available suggesting that CD disease behavior can be predicted. However, many additional studies are needed in order to confirm and compare these observations. In light of the fact that CD seems to involve numerous pathophysiologic processes and result from at least a number of disease mechanisms, likely, an algorithm combining all modalities may yield the best results for predicting the outcome of this heterogeneous disease.

## DRUG THERAPY

The other important component in the therapeutic equation is drug therapy. The treatment of CD mirrors the fact that the exact disease mechanisms are unknown and hence treatment is based on suppressing the immune system. It should be noted that due to the fact that at least in some patients CD may result from a selective immune deficiency (discussed above), future therapies may involve the opposite direction of immune enhancement. Indeed, treatment with granulocyte macrophage colony-stimulating factor (GM-CSF), an innate immune activator, was successfully used to induce remission in CD patients.^54^,^55^ These results necessitate further validation. However, despite the report of this experimental approach, the majority of treatments still apply immune suppression.

Steroids have been used for decades to treat active CD and are associated with a good effect for inducing remission.^56^ However, their use is associated with multiple side effects, both metabolic and those resulting from their immune-suppressing activity.^57^ Moreover, studies have shown that even when mucosal healing is induced by steroid treatment, the risk for subsequent disease flares is not changed.^58^ Because of this combination, steroids are used as little as possible for induction of remission only, and achievement of steroid-free remission is a major therapeutic goal.

Thiopurines have been used for many years to treat CD and were shown to be effective in maintenance of remission and steroid sparing.^59^,^60^ However, their effect on the natural history of CD is uncertain, although a recently published trial in which responding, thiopurine-treated patients were compared to non-responders demonstrated reduced rates of abdominal and perianal surgeries, albeit a mildly increased cancer rate in responders was noted.^61^ An adequate treatment response depends on proper drug dosing. Thiopurines are metabolized in the liver, and generation of adequate drug levels depends on this metabolism as well as on tissue distribution. Although the mechanism of action of thiopurines is not fully elucidated, metabolite levels have been suggested to be associated with response and can be used to aid in management of dosing and possibly liver toxicity.^62^ The main enzyme involved in generation of the active metabolite 6-thioguanin is thiopurine methyltransferase (TPMT). Genetic typing of this enzyme may aid in identifying patients at risk to develop early neutropenia.^63^

An increased risk of cancer is a major concern in thiopurine-treated patients. In a landmark study Beaugerie et al. assessed the risk of lymphoproliferative disorders according to thiopurine exposure. The median follow-up was 35 months. The study population consisted of 5867 patients receiving thiopurines, 2809 who discontinued therapy, and 10,810 controls who never received thiopurines. A total of 23 new cases of lymphoproliferative disorder were diagnosed, of which one was a Hodgkin’s lymphoma, and 22 were non-Hodgkin lymphomas. The incidence of lymphoma was 0.90 per 1000 patient-years (95% CI 0.50–1.49) for thiopurine-treated patients compared to 0.20 per 1000 (0.02–0.72) patient-years in those who discontinued treatment and 0.26 per 1000 (0.10–0.57) patient-years in those who had never received thiopurines (*P* = 0.0054). The hazard ratio of lymphoproliferative disorder between patients receiving thiopurines and those who had never received these drugs was 5.28 (2.01–13.9, *P* = 0.0007).^64^ Another risk of thiopurine therapy is for young males (<35 years), who were reported to develop lymphoproliferative disorders after EBV infection in EBV-naïve patients.^65^ Hepatosplenic T cell lymphoma is also a risk, particularly when treatment is combined with anti-TNF agents for more than 2 years in young males.^66^ Another major risk is of bone-marrow suppression which may occur already at the start of therapy in genetically susceptible hosts.^63^

Anti-TNF agents have revolutionized IBD therapy. Therapy with anti-TNF agents was shown to induce and maintain remission^67^ and was also shown to be effective for fistula closure,^68^,^69^ which is significantly superior to any other drug used for this purpose. Moreover, early treatment with anti-TNF agents (top-down approach) was shown to be superior to conventional therapy for achieving long-term mucosal healing as compared to patients treated conventionally with steroids first and immunosuppressive later on (step-up approach).^70^ Finally, anti-TNF therapy was shown to reduce hospitalizations and surgery rates.^67^,^71^ These robust results raised the possibility of changing the natural disease course and were a main driver for the development of damage and disability measurement tools mentioned above. Recent data also demonstrated that the combination of immunosuppressive therapy with anti-TNF was superior to either agent alone.^72^

Optimizing anti-TNF treatment is an evolving effort. Although there are no internationally accepted standards for therapeutic levels of drug or anti-drug antibodies, their measurement appears to correlate with response and loss of response (LOR), respectively.^73^,^74^ The antibody response is particularly heterogeneous, and many efforts are needed to fully comprehend its clinical significance.^75^

It is exactly the potent nature of these agents that elicits concerns regarding the side effects they may cause. Similar to other agents that suppress the immune system, the two main concerns are increased incidence of malignancies and serious infections. There is an inherent difficulty to measure cancer risk for patients treated by anti-TNF agents only because many are treated by combination with immunosuppressives such as thiopurines and steroids. In one study using meta-analysis, the standardized incidence ration of lymphoma in IBD patients treated by anti-TNF was estimated to be 1.7 as compared to patients treated by immunomodulation only.^76^ However, in a cohort of 6273 CD patients treated by infliximab and followed for 5 years no increased risk for lymphoma was noted. It is noteworthy that steroid treatment, narcotic analgesic treatment, and advanced age were risk factors for increased mortality, and that disease severity, steroid treatment, narcotic analgesic treatment, and infliximab were risk factors for serious infections.^77^

The use of these potent medications is further complicated by the fact that response rates are variable. Thus, in a meta-analysis the number needed to treat for induction of remission by thiopurines was five,^59^ and for maintenance of remission it was six.^60^ Response rates in individual trials ranged from 67%^78^ to as low as 30%.^72^ The response to anti-TNF agents is also not universal with approximately 20%–30% being primary non-responders^79^ and 23%–46% or 5%–13% losing response during treatment depending on the definition of LOR.^75^ The main mechanism for LOR is immunogenicity towards the anti-TNF agent, a phenomenon which can be partially prevented both by concurrent co-treatment with immunomodulators^67^ and possibly also after the occurrence of anti-drug antibodies.^80^

**Figure 1.**
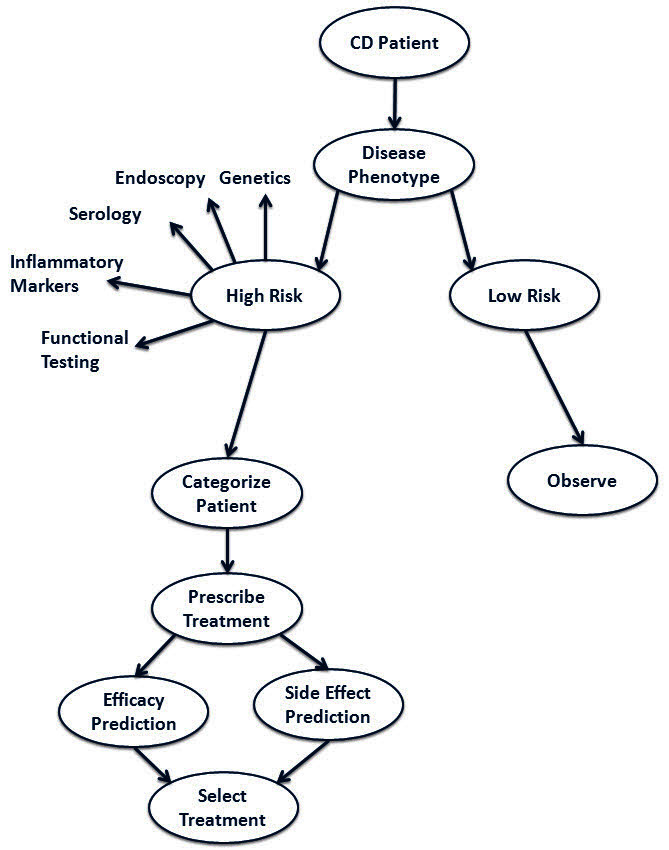
**Matching therapy to patients by foreseeing a positive response and predicting side effects.**

Taken together, the treatment of CD presents a highly complex mosaic of pathophysiologic mechanisms, disease patterns which are diverse on presentation and change during its course, uncertainty regarding response to drugs, drug interactions which can be beneficial but may also potentiate significant and even lethal side effects, and lack of proof regarding their long-term efficacy to change the course of disease. This therapeutic environment creates numerous situations in which decisions have to be taken under conditions of uncertainty, and eventually the final decision depends not only on facts, but also on the personality and subjective points of view of both the patient and physician. It is very hard to form strict treatment guidelines that will fit all CD patients, and tailoring therapy would be the only truly valid solution. Such personal treatment would have to involve matching of disease mechanisms to what appears to be a more correct definition of a specific CD syndrome variant, the ability to combine and synthesize the different prognostic tools to predict disease behavior, and the propensity of the specific variant to respond to a given therapy. Therapy itself would have to be matched to the patient by the ability to foresee a positive response and predict side effects (Figure 1). Finally, taking all the above into consideration, the algorithm will have to provide an answer to the patient: is the benefit worth the risk for me?

Little data is available to weigh treatment risks versus benefits. In a recent publication based on a single trial with a strictly defined patient population treatment success outweighed the risk of side effects.^81^ However, the specific patient population, the specific drugs analyzed, and the short follow-up period only reiterate the difficulty in obtaining such solution for the variable CD patient population. Another study demonstrated that patients place symptom control in high priority and are willing to tolerate the risks,^82^ which is an important consideration when treatment is formulated.

## CONCLUSION

With the advancement of research, the wide array of new drugs which affect different disease mechanisms, and the increasing understanding of CD pathogenesis, the relevance of various biomarkers, and the natural course and response to treatment, it is mainly a question of time before highly efficacious, safe and personal treatment is available to CD patients.

## Abbreviations:

ASCA: anti-Saccharomyces cerevisiae;
CD: Crohn’s disease;
CRP: C-reactive protein;
GM-CSF: granulocyte macrophage colony-stimulating factor;
IBD: inflammatory bowel disease;
LOR: loss of response;
OmpC: outer membrane porin C;
UC: ulcerative colitis.
